# Gastrointestinal adverse events associated with GLP-1 receptor agonists in metabolic dysfunction-associated steatotic liver disease (MASLD): a systematic review and meta-analysis

**DOI:** 10.3389/fmed.2025.1509947

**Published:** 2025-02-20

**Authors:** Xiaoyan Huang, Miaohui Wu, Baoliang Huang, Yi Zhang

**Affiliations:** ^1^Department of Endocrinology, Quanzhou First Hospital Affiliated to Fujian Medical University, Quanzhou, China; ^2^School of Pharmacy, Fujian Medical University, Fuzhou, China

**Keywords:** MASLD, GLP-1RA, adverse event, gastrointestinal reactions, meta-analysis

## Abstract

**Background:**

Metabolic dysfunction-associated steatotic liver disease, a prevalent chronic liver condition, can cause severe complications like hepatitis, cirrhosis, and hepatocellular carcinoma. In recent years, glucagon-like peptide-1 receptor agonists (GLP - 1RA) have shown unique therapeutic advantages and may become a preferred treatment for it. This meta-analysis aims to systematically examine GLP-1RA associated adverse events, providing a basis for guiding patient clinical management.

**Methods:**

We conducted a search for randomized controlled trials (RCTs) investigating the therapeutic effects of GLP-1RA in the treatment of metabolic dysfunction-associated steatotic liver disease across four databases: PubMed, Embase, Web of Science, and Cochrane Library. The search period extended from the inception of each database until December 2023. Information pertaining to various adverse events was collected as outcome measures. Statistical analysis of the results and assessment of bias risk were conducted utilizing Review Manager (version 5.4.1) software.

**Results:**

An analysis of 10 studies encompassing 960 participants revealed a significantly higher overall incidence of adverse events in the GLP-1RA group compared to the control group (OR: 2.40 [1.10, 5.26], *P* = 0.03). Subgroup analysis based on treatment duration demonstrated a higher rate of adverse events in the GLP-1RA group during follow-ups of less than 30 weeks (*P* = 0.0005, OR: 3.58 [1.75, 7.32]), but no statistical difference was observed between the two groups in follow-ups exceeding 30 weeks. There was no statistically significant difference between the two groups in adverse events leading to discontinuation (*P* = 0.29, OR: 1.47 [0.72, 2.98]). However, a notable difference was observed in gastrointestinal adverse events (*P* < 0.00001, OR: 4.83 [3.36, 6.95]).

**Conclusion:**

GLP-1RA exhibits an overall higher incidence of adverse events in the treatment of metabolic dysfunction-associated steatotic liver disease, particularly in the gastrointestinal domain. Short-term use of GLP-1RA may be associated with a greater occurrence of adverse events, underscoring the importance of educating patients on preventive measures and establishing tolerance. However, there was no statistically significant difference between the two groups in severe adverse events and adverse events leading to discontinuation, confirming the safety profile of GLP-1RA application.

## 1 Introduction

Metabolic dysfunction-associated steatotic liver disease (MASLD) is a chronic liver disease, which is primarily characterized by the abnormal accumulation of fat in the liver resulting from underlying metabolic dysfunctions. It represents a spectrum of liver diseases that are strongly associated with metabolic risk factors, such as overweight or obesity, type 2 diabetes, and other metabolic disorders. This metabolic dysfunction leads to a series of liver alterations, ranging from simple hepatic steatosis (fatty liver) to more severe forms such as MASH (Metabolic Associated Steatohepatitis), and may progress to liver cirrhosis and hepatocellular carcinoma over time (1–3). As the most common cause of steatotic liver disease, MASLD is witnessing a rapid global surge, affecting up to one-third of the world’s adult population, and posing a significant challenge to global public health (4, 5).

GLP-1RA, an exemplary drug in the treatment of type 2 diabetes, has garnered widespread attention in recent years for its potential application in the management of MASLD (6, 7). The interest in GLP-1RA is partly due to its established efficacy in improving glycemic control and promoting weight loss in patients with type 2 diabetes, which are also common features of MASLD. By mimicking the biological effects of glucagon-like peptide-1, GLP-1RA offers unique advantages in regulating blood glucose levels, improving insulin resistance, and mitigating hepatic fat accumulation, positioning it as an effective therapeutic approach for MASLD (8–11). However, despite demonstrating significant therapeutic efficacy in several clinical trials, the relatively high incidence of associated adverse reactions has constrained its widespread clinical utilization and may impact patients’ quality of life (12–14). These adverse reactions, which can include gastrointestinal symptoms such as nausea and vomiting, have led to concerns about patient tolerability and adherence to treatment. Therefore, gaining a thorough understanding of the safety profile of GLP-1RA in the treatment of MASLD, particularly regarding the nature and occurrence of adverse reactions, is crucial for a comprehensive assessment of its risk-benefit profile.

This meta-analysis is designed to systematically review the existing evidence on the safety of GLP-1RA in MASLD patients, with a focus on assessing the characteristics of adverse events. We aim to contribute to the optimization of treatment decisions and the prevention of adverse events as much as possible, thereby facilitating more effective and safer management of MASLD patients.

## 2 Methods

The research design, data collection, article composition, and result analysis in this study were conducted in accordance with the guidelines outlined in the PRISMA (2020 version) statement (15). As the data used in this study were derived from publicly available published articles and did not involve patients’ private information, ethical committee review and approval were deemed unnecessary.

### 2.1 Literature search

The preliminary data collection for this meta-analysis was independently conducted by two researchers. A comprehensive search was performed on four independent databases—PubMed, Web of Science, Cochrane Library, and Embase. The search period extended from the inception of each database to March 2024. Articles included in the search were limited to those published in English. The search strategy involved two categories of keywords: Glucagon Like Peptide 1, GLP-1, GLP 1, Glucagon-Like Peptide-1, Non-alcoholic Fatty Liver Disease, NAFLD, Metabolic dysfunction-associated steatotic liver disease, MASLD, Steatotic Liver Disease, SLD, Metabolic dysfunction-associated steatohepatitis, MASH, Nonalcoholic Fatty Liver Disease, Fatty Liver Nonalcoholic, Fatty Livers Nonalcoholic, Liver Nonalcoholic Fatty, Nonalcoholic Fatty Liver, Nonalcoholic Fatty Livers, Nonalcoholic Steatohepatitis, Nonalcoholic Steatohepatitides, Steatohepatitis Nonalcoholic. The two sets of keywords were combined using the ‘AND’ operator in accordance with Boolean logic principles to formulate the final data retrieval strategy.

### 2.2 Inclusion and exclusion criteria

#### 2.2.1 Inclusion criteria

Study Type: Randomized Controlled Trials (RCTs); Study Population: Patients diagnosed with MASLD. MASLD is defined as the presence of hepatic steatosis in conjunction with at least one cardiometabolic risk factor (CMRF), in the absence of any other discernible cause (16). CMRF encompass the following:

① BMI ≥ 25 kg/m^2^ OR waist circumference > 94 cm (male) 80 cm(female) OR ethnicity adjusted equivalent;

② Fasting serum glucose ≥ 5.6 mmol/L[100 mg/dL] OR 2-h post-load glucose levels ≥7.8 mmol/L[≥140 mg/dL] OR HbA1c ≥ 5.7%[39 mmol/L] OR type 2 diabetes OR treatment for type 2 diabetes;

③ Blood pressure ≥ 130/85 mmHg OR specific antihypertensive drug treatment;

④ Plasma triglycerides ≥ 1.70 mmol/L[150 mg/dL] OR lipid lowering treatment;

⑤ Plasma HDL-cholesterol ≤1.0 mmol/L[40 mg/dL] (male) and ≤ 1.3 mmol/L [50 mg/dL] (female) OR lipid lowering treatment.

Intervention: Experimental group received GLP-1RA, while the control group received other antidiabetic medications or a placebo; Outcome Measures: The study and statistical endpoints encompassed the occurrence of various types of adverse events, including the overall incidence of adverse events, the incidence of severe adverse events, the incidence of adverse events leading to discontinuation, and the incidence of gastrointestinal adverse events.

#### 2.2.2 Exclusion criteria

Studies published in the form of conference abstracts, reviews, animal experiments, case reports; duplicate publications; studies lacking clear conclusions; articles where full-text information cannot be obtained; studies involving participants using GLP-1RA oral formulations; and studies of poor quality, which typically include: unreasonable study design, such as non-randomized controlled trials (RCTs), lack of control groups; incomplete or inaccurate data, with missing or erroneous key outcome indicators; statistical method errors that cannot be corrected through re-analysis; with a high risk of bias.

### 2.3 Literature screening, risk of bias and data statistics

#### 2.3.1 Literature screening

The inclusion and data extraction for the study were independently performed by two researchers (MH and XY). Through initial screening (searching, reading titles and abstracts) and in-depth analysis (full-text review and internal discussions), the following data were extracted from the 10 eligible studies: the first author’s name, publication year, main study country, total number of patients involved in the trials, gender ratio, treatment regimen for the control group, and the follow-up period for the study. Any discrepancies or queries during data synthesis were resolved through internal team discussions, with final decisions made by a senior physician (YZ).

#### 2.3.2 Risk of bias

This meta-analysis employed the Cochrane Risk of Bias Assessment Tool to evaluate the risk of bias in the included studies. A risk of bias analysis was conducted for each study based on the various criteria outlined in the research guidelines (17).

#### 2.3.3 Data statistics

The summary and analysis of results were conducted using Review Manager software (version 5.4.1). Regarding the choice of the study model, if the final statistical result’s *P*-value > 0.1 and I^2^ < 50%, indicating internal consistency in the pooled studies, a fixed-effects model was chosen for the analysis. Conversely, a random-effects model was employed for analysis if *P*-value ≤ 0.1 or I^2^ ≥ 50%, suggesting significant heterogeneity among the studies. As the outcome measure was the incidence of adverse events, the analysis involved calculating the Odds Ratio (OR) for binary variables from individual trials.

In cases of substantial heterogeneity, a stepwise exclusion method and sensitivity analysis were performed to identify the source of clinical heterogeneity. If significant clinical heterogeneity was observed, subgroup analysis and funnel plots were further employed to assess publication bias.

## 3 Results

### 3.1 Literature search results

A total of 3,177 articles were initially retrieved from the databases. Among them, 982 articles were identified as duplicates. After reviewing titles, abstracts, and full texts, 2,185 articles were excluded. Finally, 10 relevant studies were included in the analysis (18–27).

### 3.2 Baseline characteristics and risk of bias

**FIGURE 1:**
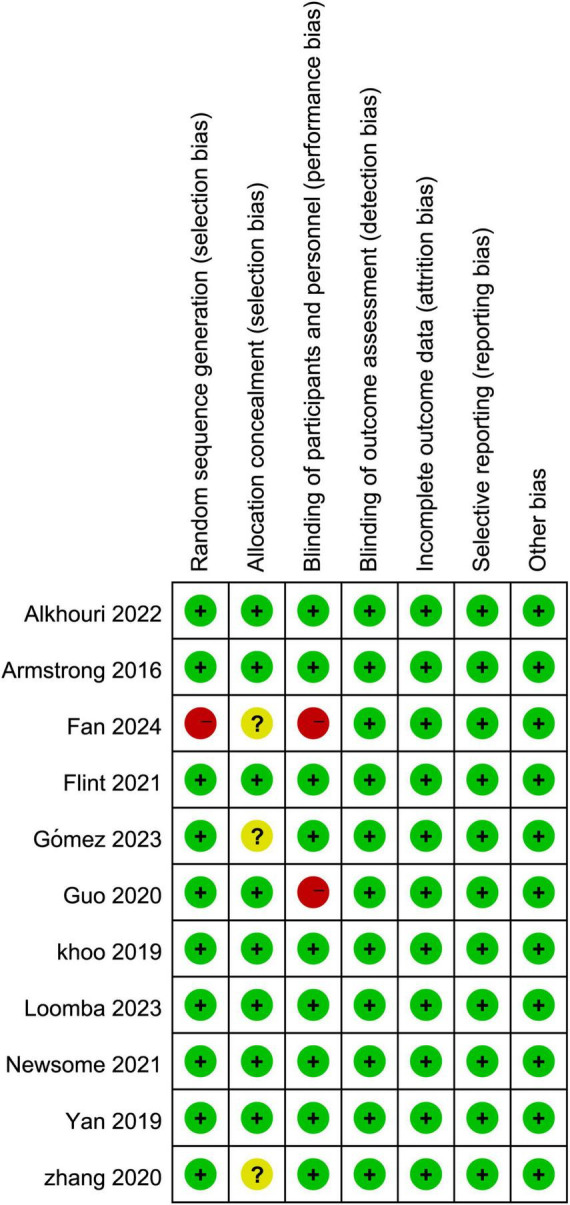
Risk assessment of bias in included literature.

A total of 10 clinical studies were included, with a combined sample size of 960 participants. Among them, 544 participants were in the intervention group, and 416 participants were in the control group. All 10 studies reported on all adverse events associated with GLP-1RA, 8 studies reported adverse events leading to treatment termination, and 6 studies reported severe adverse events related to GLP-1RA. Detailed baseline characteristics information can be found in Table 1. Overall, the included articles demonstrated a low risk of bias, indicating a high quality of evidence. Specific results are presented in Figure 1.

**TABLE 1:** Characteristics of included studies.

References	GLP-1RA drug	Dosage(mg)	Com- parison	Sample size	Age		Male (%)		BMI (kg/m^2^)		HbA1c (%)		Follow-up(wk)
					**GLP-1RA**	**Control**	**GLP-1RA**	**Control**	**GLP-1RA**	**Control**	**GLP-1RA**	**Control**	
Loomba et al. (18)	Semaglutide	2.4	Placebo	71	59.9 ± ± 7.1	58.7 ± 9.7	34	25	34.6 ± 5.9	35.5 ± 6.0	7.1 ± 1.3	7.2 ± 1.2	48
Newsome et al. (19)	Semaglutide	0.1,0.2,0.4	Placebo	319	55.8 ± 10.6	52.4 ± 10.8	51	55	35.6 ± 6.3	36.1 ± 6.6	7.3 ± 1.2	7.3 ± 1.2	72
Gómez et al. (20)	Semaglutide	1	Efinopeg-dutide	145	48.1 ± 11.0	50.9 ± 10.9	54	56	35.2 ± 5.7	33.5 ± 5.0	5.9 ± 0.5	5.9 ± 0.6	32
Armstrong et al. (21)	Liraglutide	1.8	Placebo	52	50.0 ± 11.0	52.0 ± 12.0	69	50	34.2 ± 4.7	37.7 ± 6.2	5.9 ± 0.7	6.0 ± 0.9	48
Flint et al. (22)	Semaglutide	0.4	Placebo, Sitagliptin	67	59.5 ± 10.1	60.5 ± 8.5	68	73	34.2 ± 5.3	33.5 ± 5.0	6.6 ± 0.8	6.5 ± 0.8	72
Yan et al. (23)	Liraglutide	1.8	Insulin	75	43.1 ± 9.7	45.6 ± 7.6	71	58	30.1 ± 3.3	29.6 ± 3.5	7.8 ± 1.4	7.7 ± 0.9	26
Khoo et al. (24)	Liraglutide	3	Non-drug	30	38.6 ± 8.2	43.6 ± 9.9	100	87	34.3 ± 3.9	32.2 ± 3.2	5.7 ± 0.5	6.1 ± 0.8	52
Zhang et al. (25)	Liraglutide	1.2	Pioglitazone	60	50.2 ± 11.5	51.5 ± 12.1	43	50	27.6 ± 5.2	27.1 ± 3.8	8.1 ± 2.0	8.1 ± 1.7	24
Guo et al. (26)	Liraglutide	1.8	Placebo, Insulin	91	53.1 ± 6.3	52.0 ± 8.7	51	60	29.2 ± 4.2	28.3 ± 3.8	7.5 ± 1.3	7.4 ± 0.9	26
Fan et al. (27)	Beinaglutide	0.1	Non-drug	50	47.2 ± 13.0	52.8 ± 15.2	52	60	30.5 ± 4.0	30.1 ± 4.7	6.8(6.2, 7.7)	6.7(6.2, 7.1)	24

BMI, Body Mass Index; HbA1c, Glycated hemoglobin A1c; GLP-1RA, Glucagon-Like Peptide-1 Receptor Agonist.

### 3.3 Total AEs

**FIGURE 2:**
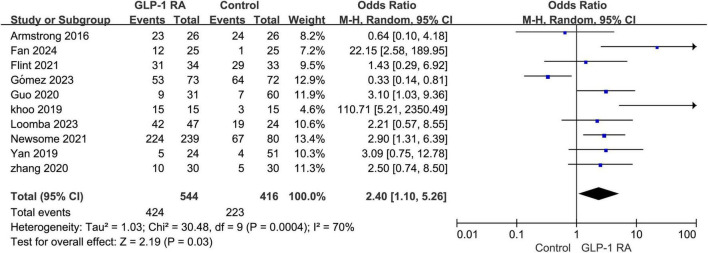
Total incidence of adverse events in GLP-1RA compared to the control group.

The meta-analysis of the 10 included studies revealed that patients undergoing GLP-1RA treatment had an overall higher incidence of adverse events compared to the control group. The results showed a statistically significant difference with an odds ratio (OR) of 2.40 [1.10, 5.26], and a *P*-value of 0.03 (Figure 2).

### 3.4 Follow-up < 30 week VS > 30 week

**FIGURE 3:**
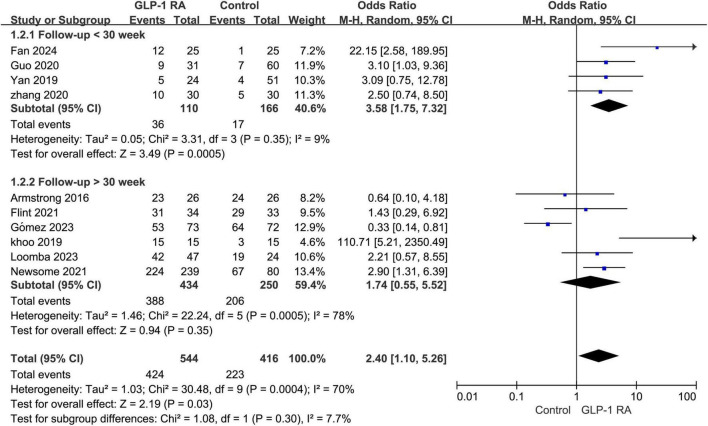
Subgroup analysis of GLP-1RA compared to control group with a treatment period greater than or less than 30 weeks.

Subgroup analysis was conducted on the statistical results of all adverse events, stratifying the analysis based on whether the treatment and follow-up duration exceeded 30 weeks. The results indicated that when the treatment follow-up period was less than 30 weeks, GLP-1RA treatment was associated with a higher incidence of adverse events compared to the control group, and this difference was statistically significant (*P* = 0.0005, OR: 3.58 [1.75, 7.32]). However, when the treatment follow-up period exceeded 30 weeks, the incidence of adverse events with GLP-1RA did not show statistical significance compared to the control group (*P* = 0.35, OR: 1.74 [0.55, 5.52]) (Figure 3).

### 3.5 Serious adverse event VS mild to moderate adverse event

**FIGURE 4:**
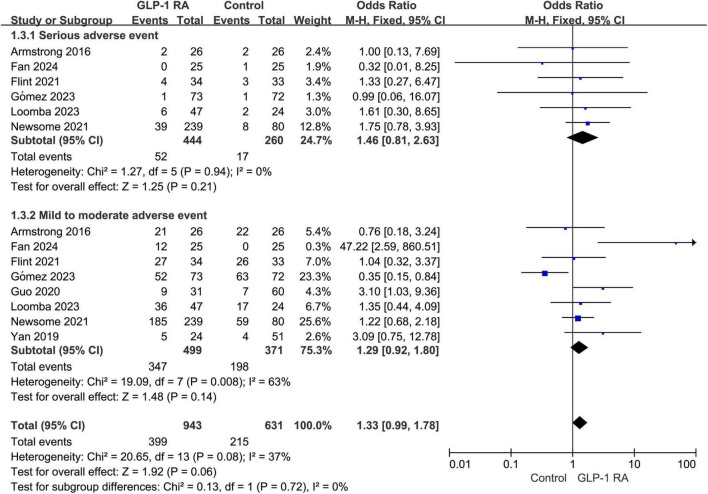
Subgroup analysis of GLP-1RA compared to control group with different levels of adverse events.

Subgroup analysis was conducted on the statistical results of all adverse events, stratifying the analysis based on whether the adverse events were categorized as severe. The results indicated that there was no statistically significant difference in the severity of adverse events between the two groups (*P* = 0.06, combined OR: 1.33 [0.99, 1.78]) (Figure 4).

### 3.6 Adverse events leading to drug discontinuation

**FIGURE 5:**
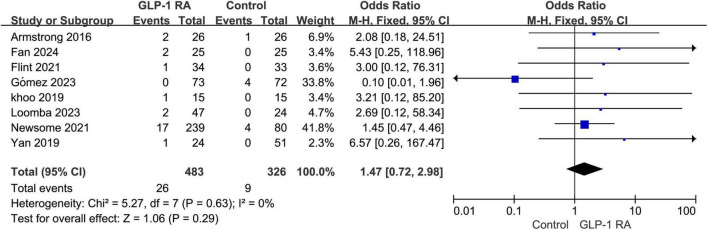
Incidence of discontinuation due to adverse events in GLP-1RA versus control group.

A total of 8 articles addressed discontinuation of treatment due to adverse events. According to the forest plot analysis, the *P*-value was 0.29, with an odds ratio (OR) of 1.47 [0.72, 2.98]. The results indicate that there is no statistically significant difference in the rate of adverse events leading to discontinuation between the GLP-1RA group and the control group (Figure 5).

### 3.7 Gastrointestinal adverse events between GLP-1RA and control group

**FIGURE 6:**
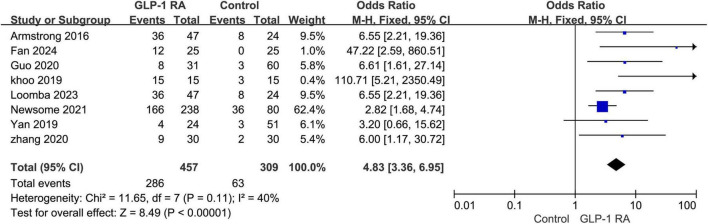
Incidence of gastrointestinal adverse events in GLP-1RA versus control group.

A total of 8 studies provided specific data on gastrointestinal adverse events. Upon summarizing the data for GLP-1RA and the control group, a statistically significant difference in the incidence of gastrointestinal adverse events between the two groups was observed (*P* < 0.00001, OR: 4.83 [3.36, 6.95]). The I^2^ value was 40%, indicating internal consistency among the studies. The results were presented using a fixed-effects model (Figure 6).

### 3.8 Types of gastrointestinal adverse events with GLP-1RA

**FIGURE 7:**
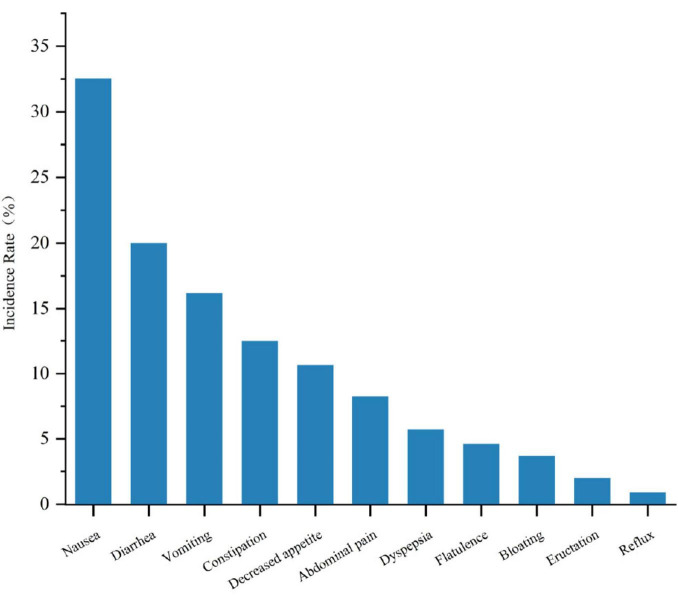
Comparison of the incidence rates of different gastrointestinal adverse reactions in GLP-1RA therapy for MASLD.

In the treatment of MASLD with GLP-1RA, a spectrum of gastrointestinal adverse events was observed, with an overall mean incidence rate of 58.9% for these events. The specific incidence rates are detailed in Figure 7. Nausea was the most frequently reported adverse event, affecting 32.54% of patients. Diarrhea and vomiting were also common, with incidence rates of 19.99% and 16.18%, respectively. Constipation and decreased appetite were noted in 12.5% and 10.66% of patients, respectively. Abdominal pain and dyspepsia occurred in 8.27% and 5.7% of patients, respectively. Less common events, including flatulence (4.6%), bloating (3.68%), eructation (2.02%), and reflux (0.92%), were also observed.

### 3.9 Publication bias

**FIGURE 8:**
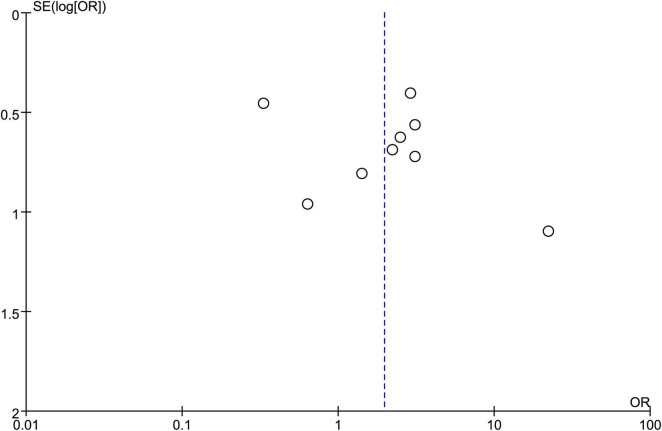
Funnel plot of included studies.

After consolidating data from the 10 included articles, observation of the funnel plot revealed a generally symmetrical distribution of points on both sides, indicating the absence of publication bias (Figure 8).

## 4 Discussion

This study systematically reviewed the adverse events associated with GLP-1RA therapy for MASLD. Despite demonstrating significant advantages in treatment efficacy, GLP-1RA revealed a higher overall incidence of adverse events, thereby limiting its extensive clinical application. These findings are consistent with the results observed in most past clinical trials, highlighting the inherent contradiction and complexity in the risk-benefit profile of GLP-1RA therapy for MASLD (28–31).

The subgroup results of the meta-analysis revealed that, during the treatment follow-up period of less than 30 weeks, the incidence of adverse events in the GLP-1RA group was significantly higher than that in the control group. This suggests that short-term treatment with GLP-1RA may lead to a higher occurrence of adverse events, particularly in patients who are newly exposed to such medications and have not yet developed tolerance. However, when the treatment follow-up period exceeded 30 weeks, there was no statistically significant difference in the incidence of adverse events between the GLP-1RA group and the control group. This implies that the long-term use of GLP-1RA is safer, and in clinical practice, it is advisable to employ extended dosing regimens whenever possible.

Subgroup analysis of adverse events of varying severity did not reveal any statistically significant differences between the GLP-1RA group and the control group, indicating that the heterogeneity between the two groups does not stem from the severity of adverse events. However, given the proximity of the results’ *p*-values to the significance threshold and the diverse nature of adverse events, we posit that further research with a larger sample size and more comprehensive data is needed to verify whether there is an association between the severity of adverse events and the two groups.

Despite clinical trials reporting instances of discontinuation of GLP-1RA treatment due to adverse events (32–35), the results of the meta-analysis did not reveal any statistically significant differences between the GLP-1RA group and the control group in this regard. This suggests that, although GLP-1RA may lead to certain adverse events, the occurrence of patient-initiated discontinuation in response to these events is not significant. Considering the absence of statistically significant differences in severe adverse events between the two groups, we infer that the safety profile of GLP-1RA remains relatively high. However, given the overall higher incidence of adverse events associated with GLP-1RA, we conducted a subgroup analysis focusing on the most commonly reported gastrointestinal adverse events in previous GLP-1RA studies (36, 37). The analysis revealed a significant statistical difference between the GLP-1RA treatment group and the control group, indicating that gastrointestinal adverse events are indeed a relatively prominent concern in GLP-1RA therapy. The low internal heterogeneity observed after grouping suggests that gastrointestinal adverse events may be a major contributing factor to the overall adverse event profile.

In our analysis of the included studies, we aggregated the adverse events associated with GLP-1RA treatment. Subgroup analysis of the GLP - 1RA group revealed a relatively high overall mean incidence of gastrointestinal adverse events, which was 58.9%. The incidence of discontinuation due to adverse events was 4.41%. Nausea, as the most prevalent adverse event, affected up to 32.54% of the patients. This finding highlights the necessity of in-depth investigation into the influencing factors of adverse events. Due to the limited number of included studies and the lack of comprehensive raw data, it was difficult to perform a standardized correlation analysis. However, after attempting a preliminary analysis of the combined data, we identified potential associations. After considering the sample size weights, we performed a correlation analysis on patient age, gender, BMI, glycated hemoglobin levels, drug dosage, and the incidence of various adverse events. The results showed a significant positive correlation between the incidence of gastrointestinal events and the discontinuation rate due to adverse events (*P* < 0.01). Additionally, a higher total incidence of adverse events was associated with a higher incidence of severe adverse events (*P* < 0.05). Notably, a higher proportion of female patients was also related to an increased incidence of gastrointestinal events and discontinuation due to adverse events (*P* < 0.05). These correlation analysis results indicate that early intervention for adverse events and high-risk populations may bring clinical benefits. For example, early intervention for patients at high risk of developing gastrointestinal adverse events, or implementing more preventive strategies for female patients, might improve patients’ treatment adherence.

Given the significant impact of gastrointestinal adverse events on patients, several strategies can be implemented to effectively manage these issues. First, gradually increasing the dose of GLP - 1RA can help patients better tolerate the treatment and reduce the incidence of gastrointestinal adverse events. Second, educating patients about the potential side effects of GLP - 1RA, and informing them that adverse reactions usually subside as their body adapts, while providing them with strategies to manage these symptoms (such as dietary adjustments, adjusting eating frequency, and appropriate exercise) has been shown to alleviate gastrointestinal discomfort (38–40). Furthermore, providing supportive care measures, including antiemetics, acid suppressants, or antidiarrheals, can help control symptoms and enhance patient comfort. Additionally, regular monitoring of patients during treatment, especially during the initial treatment period, is crucial for early identification and resolution of adverse events. Early intervention can prevent symptom exacerbation and reduce the likelihood of treatment discontinuation.

For patients with persistent symptoms, temporary medication breaks or “drug holidays” may be considered to facilitate symptom resolution. In cases where adverse events are intolerable, alternative therapeutic strategies must be promptly explored. This may involve adjustments to the treatment regimen, such as switching to a different GLP - 1RA or combining it with other medications with a lower risk of gastrointestinal side effects. Numerous studies have demonstrated differences in the incidence of gastrointestinal adverse events among various GLP - 1RAs (41–43). For example, tirzepatide may cause fewer such events compared to semaglutide (44), selecting a GLP - 1RA with better gastrointestinal safety for high - risk patients might be a beneficial strategy.

The strengths of this study lie in the inclusion of a substantial number of high-quality randomized controlled trials, encompassing overall adverse events, follow-up duration, severity, discontinuation rates, and specific gastrointestinal adverse events. This approach has enhanced the credibility and representativeness of the meta-analysis to a certain extent. Additionally, the utilization of various subgroup analyses, examining factors that may influence adverse events, contributes to a more comprehensive evaluation for clinical decision-making.

However, the limitations of this study should be acknowledged. We restricted our search to articles published in English, potentially introducing language bias. Moreover, despite incorporating data from 10 clinical studies, the overall sample size was relatively small, which may impact the stability and generalizability of the results. Future research efforts should consider including larger sample sizes to further validate the study findings. Additionally, within the limited scope of available studies, the inclusion of diverse control interventions has hindered a more detailed exploration of varying doses of the same treatment regimen. This limitation underscores the need for future trials to provide more robust evidence in this regard.

## 5 Conclusion

In summary, GLP-1RA demonstrates a relatively high level of safety in the treatment of MASLD. There is no statistically significant difference compared to the control group in terms of severe adverse events and adverse events leading to discontinuation. Further analysis reveals a higher overall incidence of adverse events associated with GLP-1RA, primarily concentrated in the early stages of treatment and related to the gastrointestinal system. Therefore, when using GLP-1RA in the treatment of MASLD patients, overall safety is favorable, but monitoring patients’ tolerance to early treatment and their gastrointestinal adverse reactions is crucial.

## Data Availability

The original contributions presented in this study are included in this article/supplementary material, further inquiries can be directed to the corresponding author.
